# Bioprospecting for acidophilic lipid-rich green microalgae isolated from abandoned mine site water bodies

**DOI:** 10.1186/2191-0855-4-7

**Published:** 2014-03-26

**Authors:** Joseph K Eibl, Jason D Corcoran, Gerusa N A Senhorinho, Kejian Zhang, Nekoo Seyed Hosseini, James Marsden, Corey A Laamanen, John A Scott, Gregory M Ross

**Affiliations:** 1 Bharti School of Engineering, Laurentian University, Sudbury, ON, Canada; 2 Biomolecular Sciences Program, Laurentian University, Sudbury, ON, Canada; 3 Northern Ontario School of Medicine, 935 Ramsey Lake Rd, Sudbury, ON P3E 2C6, Canada

**Keywords:** Microalgae, Biofuel, FAME, Bioprospecting

## Abstract

With fossil fuel sources in limited supply, microalgae show tremendous promise as a carbon neutral source of biofuel. Current microalgae biofuel strategies typically rely on growing high-lipid producing laboratory strains of microalgae in open raceways or closed system photobioreactors. Unfortunately, these microalgae species are found to be sensitive to environmental stresses or competition by regional strains. Contamination by invasive species can diminish productivity of commercial algal processes. A potential improvement to current strategies is to identify high-lipid producing microalgae, which thrive in selected culture conditions that reduce the risk of contamination, such as low pH. Here we report the identification of a novel high-lipid producing microalgae which can tolerate low pH growth conditions. Lig 290 is a *Scenedesmus spp*. isolated from a low pH waterbody (pH = 4.5) in proximity to an abandoned lignite mine in Northern Ontario, Canada. Compared to a laboratory strain of *Scendesmus dimorphus*, Lig 290 demonstrated robust growth rates, a strong growth profile, and high lipid production. As a consequence, Lig 290 may have potential application as a robust microalgal species for use in biofuel production.

## Introduction

Global fossil fuel stores are in limited supply and a concerted effort has been made to identify biofuels that might be viable alternatives to fossil fuels (Larkum et al. 2011; Williams 2007). Historically, most forms of biofuel were derived from crops grown on arable land. Unfortunately, the use of agricultural land to grow energy stores is not seen to be a sustainable solution given the increasing global demand for food crops (Delrue et al. 2012). Microalgae have been identified as a viable source of biofuel that does not take up arable land and could one day supplement, or even replace, fossil fuels for many industrial uses (Chisti 2008; Delrue et al. 2012).

Microalgae are unique photosynthetic microorganisms which have the ability to convert solar energy into biomass (Larkum et al. 2012). Compared to traditional biofuel plant feed stocks, microalgae have the advantage of growing at much greater densities per unit area, can be grown on non-arable land, and can be grown in fresh water, brown water, saline or oceanic environments (Chisti 2007; Gong and Jiang 2011; Larkum et al. 2012).

Under the appropriate environmental conditions, some microalgal species produce concentrated stores of triacylglyceride (Halim et al. 2012). Using the chemical process of transesterification, phospholipids can be easily cleaved and converted into fatty acid methyl esters (FAME) which are a versatile form of biodiesel (Chisti 2007; Cirulis et al. 2012). Microalgae-based biofuels are recognized as a viable supplement and/or alternative to fossil fuels for many industrial uses. In fact, microalgae biofuel has the potential to remedy several challenges currently associated with fossil fuels and first-generation biofuels (Gong and Jiang 2011). For example, microalgal-based biofuels have the advantages of being: i) a carbon neutral fuel source, ii) a cleaner burning alternative to fossil fuels, iii) grown on non-arable land, and iv) able to utilize and mitigate the CO_2_ in industrial off-gas, unlike first and second-generation biofuels (Halim et al. 2012).

Currently, the strategy for large scale algal production utilizes characterized laboratory strains of algae which can rapidly grow to a dense biomass. There are approximately 60 species which are well characterized in the biofuel field (Chisti 2007; Doan et al. 2011). Typically, algae growing in log phase will have relatively low lipid content (5-10%) (Pan et al. 2011). Once the culture has reached a satisfactory density, a stress or trigger can be applied to the culture to induce a change from photosynthetic metabolism to lipid metabolism (Halim et al. 2010; Kaur et al. 2011; Pan et al. 2011). Common lipid metabolism inducing triggers can include nutrient-, pH-, metal-, or temperature-based stresses (Lardon et al. 2009). Under optimal stress conditions, several green microalgae species have been reported to produce 30-50% lipid as fraction of dry cell weight (Chisti 2007; Halim et al. 2010).

While mass microalgae production has vastly improved in the last decade, open pond technologies remain the most economical and widely used strategy for biofuel production (Chen et al. 2010). However, open pond culturing has yet to overcome the challenge of invasive species contamination which can result in diminished productivity of the process (Chen et al. 2010). A potential improvement to current strategies is to identify a robust high-lipid producing microalgae strain which thrives in selected culture conditions that discourage invasive species (Do Nascimento et al. 2012; Larkum et al. 2012). Many selective culturing practices existing in the field of microbiology can be utilized in algal biotechnology; for example, *Spirulina* can be cultured in high salinity (Wang et al. 2013). In freshwater environments, perhaps the most accessible is the ability to modulate the pH of culture media to mitigate contamination (Chen et al. 2010). However, few if any commercial microalgae processes employ this strategy due to the common use of well characterized strains which typically grow at neutral pH (Chen et al. 2010). Thus, the field of microalgal-based biofuel production may benefit from the identification of an acid-tolerant high-lipid producing strain of microalgae.

Extreme environments do exist where it may be possible to identify acid-tolerant microalgae. For example, the province of Ontario, Canada has more than 5000 abandoned mine sites according to the Abandoned Mines Information System (AMIS; Ontario Ministry of Northern Development and Mines). Historically, many of these mines have had little if any environmental remediation following mine closure, and sulfur rich tailings producing a variety of low pH water bodies exist in proximity to the abandoned mine site (Muzik 1982). Therefore, it is possible that acid-tolerant lipid rich algae could be isolated from such an environment.

In this study, we bioprospected water bodies in proximity to an abandoned lignite mine in Northern Ontario, Canada to look for stress-resistant high-lipid producing green microalgae. Multiple water bodies were sampled in proximity to the Onakawana lignite mine and recovered environmental green microalgae samples cultured. Green microalgae were characterized based on stress-resistance and lipid-production as benchmarked against a standard laboratory strain of *Scenedesmus dimorphus*. The results of this study identified the environmental isolate Lig 290, a stress-resistant high-lipid producing green microalgae which demonstrates desirable growth characteristics for biofuel cultivation.

## Materials and methods

### Mapping

Maps were rendered using ArcMap 10.0 (Esri, Toronto, ON, Canada). Abandoned mine sites were plotted from global positioning system coordinates provided by the Ontario Ministry of Natural Resources (Sudbury, ON, Canada). Sampling locations were obtained using a hand held GPS unit (Garmin, Olathe, KS, USA).

### Environmental sampling

Aerial sampling was performed using a Bell 206 helicopter and at each site 500 mL of water was collected from the water surface, and logged with GPS location, time and date. Upon return to the lab, environmental water chemistry was analyzed and the results were recorded. Water chemistry analysis was performed by Xstrata Process Support (Falconbridge, ON, Can).

### Microscopy and algal speciation

Algae were identified via morphological assessment (Bellinger and Sigee 2010). The wild sample was subject to purification by serial dilution and then transferred to Bold’s Basal Medium agar plates (Bold and Wynne 1985) solidified with 1.5% (w/v) of bacteriological agar. Algal identification to the genus level was performed using morphological analysis based on the Key to the More Frequently Occurring Freshwater Algae described by Bellinger and Sigee (Bellinger and Sigee 2010). Morphological observation was performed using brightfield microscopy with an AMG EVOS XL light microscope equipped with a 100× objective (Bothwell Washington, USA). Algae were identified to the *Scenedesmus* genus based on the observed lateral quartet coenobia morphology. Lig 290 has been deposited in the World Datacenter for Microorganisms.

### Culture conditions

Single colonies of Lig 290 were obtained and cultured in a modified CHU 10 medium (Bold and Wynne 1985) at 25°C. Isolated cultures were grown under photosynthetic light (Infors, Montreal, Quebec, Canada; 2700 Lux/~135 W/m^2^) in cycles of 12 hr light and 12 hr dark while continuously agitated at 125 rpm. In conditions where pH levels were modified from neutral pH, adjustments were performed every second day using concentrated sulfuric acid. The modified media had carbon sources removed to make it suitable for non-xenic culturing beyond basic nutrient needs. This modified solution reflects environmental micronutrient levels. The media was confirmed to be stable at pH 3–8. No precipitation was observed across this pH range.

### Cell counting

Algae cell concentrations were calculated by pipetting a 20 μL sample of each culture onto a Cellometer counting chamber (Nexcelom Bioscience, Lawrence, MA, USA) and analyzed using a Cellometer Auto X4 Cell Counter (Nexcelom Bioscience, Lawrence, MA, USA).

### Flow cytometry

Flow cytometry was utilized for lipid analysis by measuring the single cell fluorescence of cells stained with Nile Red, a lipophilic dye, as a relative lipid indicator. A 100 μg/mL stock solution of Nile Red in acetone (Sigma, Oakville, Ontario, Canada) was made and stored as outlined by Cirulis et al. ( 2012).

Microalgal samples were stained with Nile Red at a concentration of 1 μg/mL in 2 mM phosphate buffer (pH 5.0) and allowed to incubate for 25 minutes prior to analysis. Relative Nile Red fluorescence was analyzed using a BD FACSCanto II flow cytometer (BD Biosciences, San Jose, CA, USA). Nile Red (ex: 488 nM; em: 585 nm), chlorophyll (ex: 488 nM; em: 780 nm), and single cell fluorescence were measured according to the methods outlined in Cirulis et al. ( 2012). Data was collected for a total of 10,000 events. Flow cytometry data was further analyzed using BD FACSDiva Software v5.0.1 (BD Biosciences, San Jose, CA, USA). A similar gating strategy was used as in Cirulis et al. ( 2012). The means and standard deviation of each population were averaged across triplicate measures.

### Total lipid analysis

Lipid extraction was performed using a modification of the method described by ( Folch et al. 1957). Freeze-dried algae samples (100 mg) were mixed with 3 mL of chloroform:methanol (2:1 v/v) in a centrifuge tube and then sonicated using a Sonic Dismembrator Model 500 (Fisher Scientific, Ottawa, Ontario, Canada) for approximately 1 minute. The samples were centrifuged using an Allegra X-15R Centrifuge (Beckman, Palo Alto, CA, USA) and the solvent was removed to a weighed vial. Extraction of biomass was repeated three times and the resulting solvent was combined. The combined extract was dried by blowing a stream of nitrogen gas and the mass of the lipid obtained determined gravimetrically.

### Direct transesterification

Direct transesterification was performed as described in Velasquez-Orta et al. ( 2012). Freeze-dried algae samples (100 mg) were placed in a glass tube and mixed with 2 mL methanol:hexane (1:1 v/v). Sodium methoxide (100 μL) was then added to begin the reaction. The reaction mixture was heated at 80°C for 1 hour and kept well-mixed. After the reaction was complete, 0.5 mL HCl was added to neutralize the catalyst and stop the reaction. Samples were centrifuged and the upper hexane layer containing the FAME extract was transferred to a gas chromatography vial for analysis (Velasquez-Orta et al. 2012).

### Gas chromatography

The composition of FAME was analyzed via gas chromatography (Thermo Trace 1300, Thermo Canada, Ottawa, Ontario, Canada) that was equipped with a flame-ionization detector (FID) and a SGE SolGel-Wax capillary column (30 m × 0.25 mm × 0.25 μm) (Canadian Life Sciences, Peterborough, Ontario, Canada). FAME samples were dissolved in hexane with C17:0 added as internal standard. Helium was used as carrier gas at a constant flow rate of 1.6 mL/min. Standard split/splitless injection was used with a split ratio of 80 and an injector temperature of 250°C. The column temperature was from 140°C to 240°C at 4°C/min. Detector temperature was 280°C. A FAME Mix C4-C24 was used as an external standard to identify the retention time for FAME peaks. Peak areas were used to quantify each FAME relative to the internal standard.

## Results

**Figure 1:**
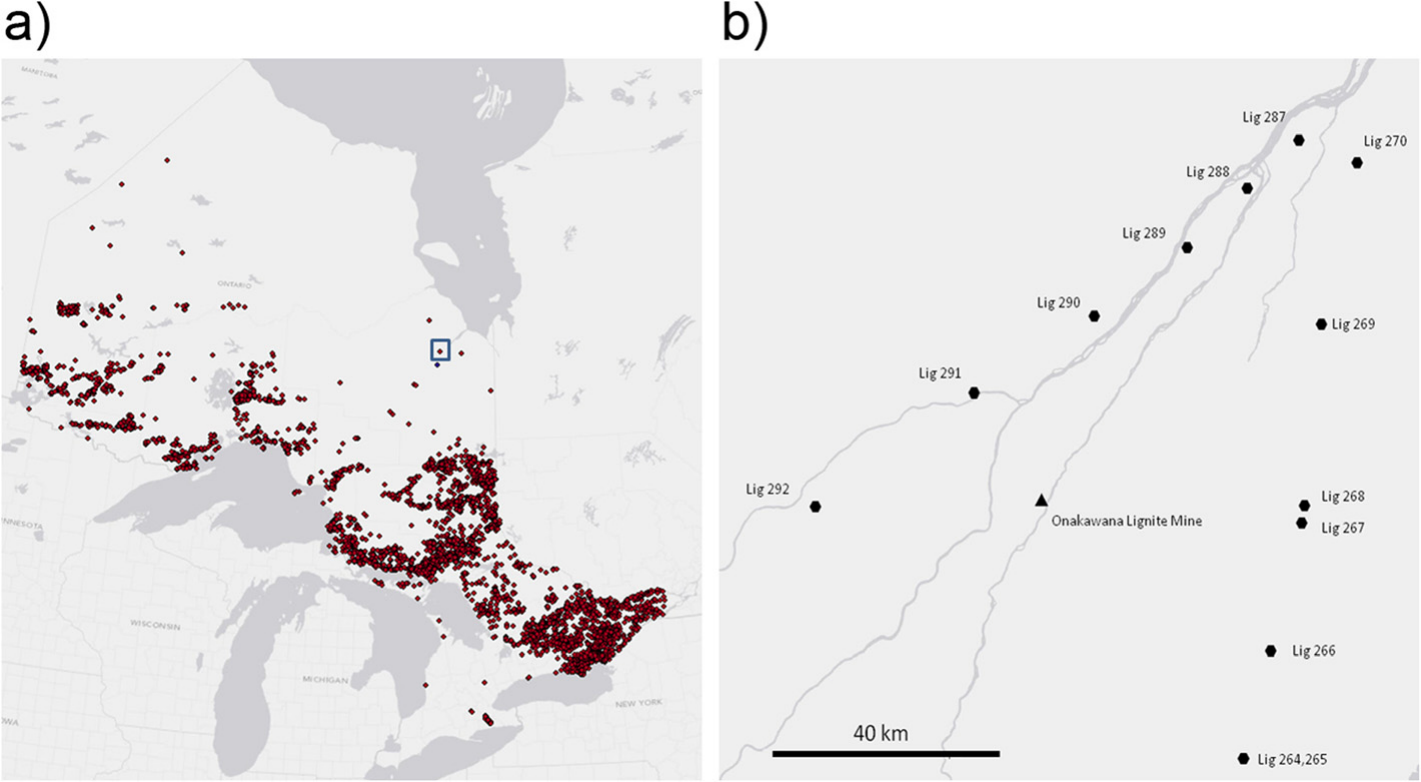
**Identification of stressed environments from the Abandoned Mines Information System. a)** The Abandoned Mines Information System is a dataset maintained by the Ontario Minister of Natural Resources. The dataset identifies 5200 abandoned mine sites across Ontario. The Onakawana site (highlighted by a blue box) was the only identified lignite mine in the province. Additionally, the mine site was abandoned without a closure plan. **b)** Twelve waterway samples (each identified by a hexagon) were collected in proximity to the Onakawana mine site (triangle).

Ontario covers one million square kilometers of Canada, of which approximately 61% is composed of mineral rich pre-cambrian rock. Due to this abundant store of minerals, Ontario has been a mining hub for over a century. As a legacy of its mining history, Ontario has over 5000 abandoned mines according to the Abandoned Mines Information System (AMIS; Ontario Ministry of Northern Development and Mines), which are illustrated Figure 1a. Government regulations have been implemented to mitigate the environmental impact resulting from mining activities. However, strict environmental regulations have only become stringent in the last two decades and thus mines pre-1990 had few, if any, closure strategy requirements.

We hypothesized that bioprospecting areas of historic mining activity could yield microalgal species which have evolved to be tolerant to extreme environments, in particular low pH, and could act as a productive source of biomass in a typical open pond production setting. To achieve this we used the AMIS to screen 5200 abandoned mine sites. As a result, twenty seven mines were identified as being classified as *Potential Concerns Regarding the Receiving Environments*.

In order to identify extremeophile microalgae that are tolerant of low pH, we queried the AMIS database to identify any lignite mines (brown coal). Lignite is typically mined from soil which is rich in sulfur, which can give rise to low pH water bodies (Muzik 1982; Shang and Scott 2011). According to the AMIS database, the Onakawana mine (located at 81.433 longitude and 48.419 latitude) is the only abandoned lignite mine registered in Ontario. Lignite was mined there during the early 1980’s and no mine closure plan was filed or reported (Muzik 1982). Thus, we expected there to be a high probability of identifying low pH water bodies in proximity to the mine site where acid-tolerant high-lipid producing microalgae may be found.

Due to the remote nature of the Onakawana lignite mine, an aerial sampling strategy was employed and twelve samples were collected in proximity to the mine. Figure 1b illustrates the sampling locations. At each location, 500 ml of water was sampled and returned to the laboratory for culturing and chemical analysis.

**Figure 2:**
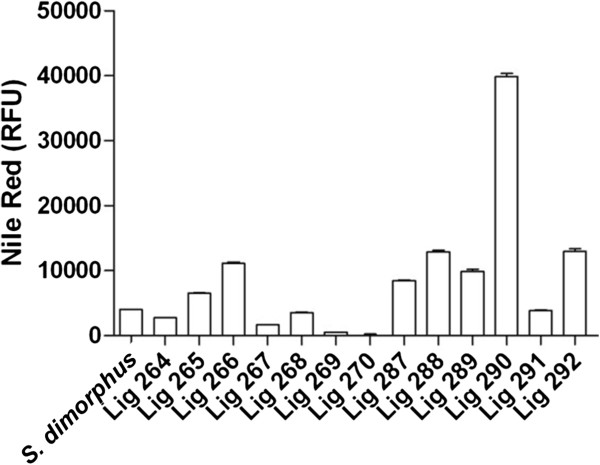
**Screening of environmental samples for high-lipid producing microalgae.** Analysis of Nile Red stained samples via flow cytometry identified Lig 290 as a high lipid producing sample. Experimental results are based on a mean analysis of 10,000 events and error bars represent standard error of the mean.

To identify algal species that could be high-lipid producing algae, we employed a lipophilic Nile Red screening strategy as a means of quantifying relative lipid levels. Microalgae collected from the wild were stained and analyzed via flow cytometry (Cirulis et al. 2012). Figure 2 illustrates Nile Red screening of environmental samples collected in proximity to the Onakawana mine. Interestingly, the two samples having the highest Nile Red signal (Lig 266 and Lig 290) were isolated from a low pH environment (pH <5), and both of these samples produced higher signals than a laboratory strain *Scenedesmus dimorphus*) commonly used in biofuel studies. Due to Lig 290 producing the highest lipophilic signal and being identified from a low pH environment, we chose to isolate the strains of microalgae in the hope of identifying a high lipid variety. Thus, we selected Lig 290 as a candidate sample to characterize as a potential high-lipid producing pH tolerant microalgae strain, which could be used in biofuel production.

**Figure 3:**
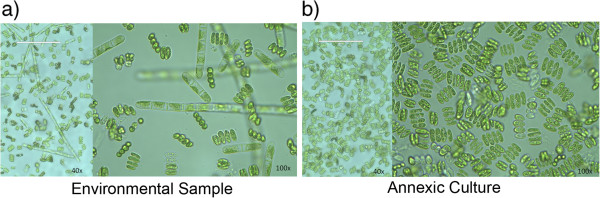
**Visual assessment of Lig 290.** An environmental sample direct from location Lig 290 and an axenic culture of Lig 290 were visualized via bright field microscopy. **a)** The environmental sample contained a communities of unicellular, multicellular and filamentous green microalgae. **b)** An isolated *Scendesmus spp.* was isolated from the environmental sample. Images are 40× and 100× objective magnification. White scale bars provided at the top left are 100 μm.

Microscopic assessment of Lig 290 indicated a community of microorganisms including filamentous and unicellular green microalgae (Figure 3a). Of the photosynthetic organisms, morphological analysis indicated that *Scenedesmus* was the most prevalent genus of green microalgae. An axenic culture was obtained using a standard agar streaking technique. Figure 3b illustrates the resulting Lig 290 axenic culture.

To assess if Lig 290 is suitable for use as a biofuel producing strain of microalgae, we characterized the growth rate over the course of 35 days. Over this period, cultures demonstrated high productivity at pH 7 and pH 4, and were still able to grow at pH 3 after a relative period of adaptation. This pH adaptation phenomenon has been characterized in other *Scenedesmus* species (Jian et al. 2012); however, culturing microalgae at low pH conditions for the purposes of biofuel production is not well studied.

The growth profile of Lig 290 is presented in Table 1. During the log phase of growth, Lig 290 reached a projected productivity of approximately 44 mg/L/d at pH 7; 52 mg/L/d at pH 4; and 32 mg/L/d at pH 3. These results indicate its suitability for cultivation in acidic conditions.

**Table 1:** Growth profile of Lig 290 in acid culture conditions

**pH**	**Days in culture**	**Initial biomass (mg/L)**	**Duration of lag phase**	**Specific Growth rate in log phase (1/d)**	**Biomass productivity in log phase (mg/L/d)**	**Projected biomass after day 35 (mg/L)**
7	35	42.6	15	0.07	43.86	1023.16
4	35	42	17	0.08	51.85	1059.82
3	35	42.2	21	0.16	32.32	506.58

**Figure 4:**
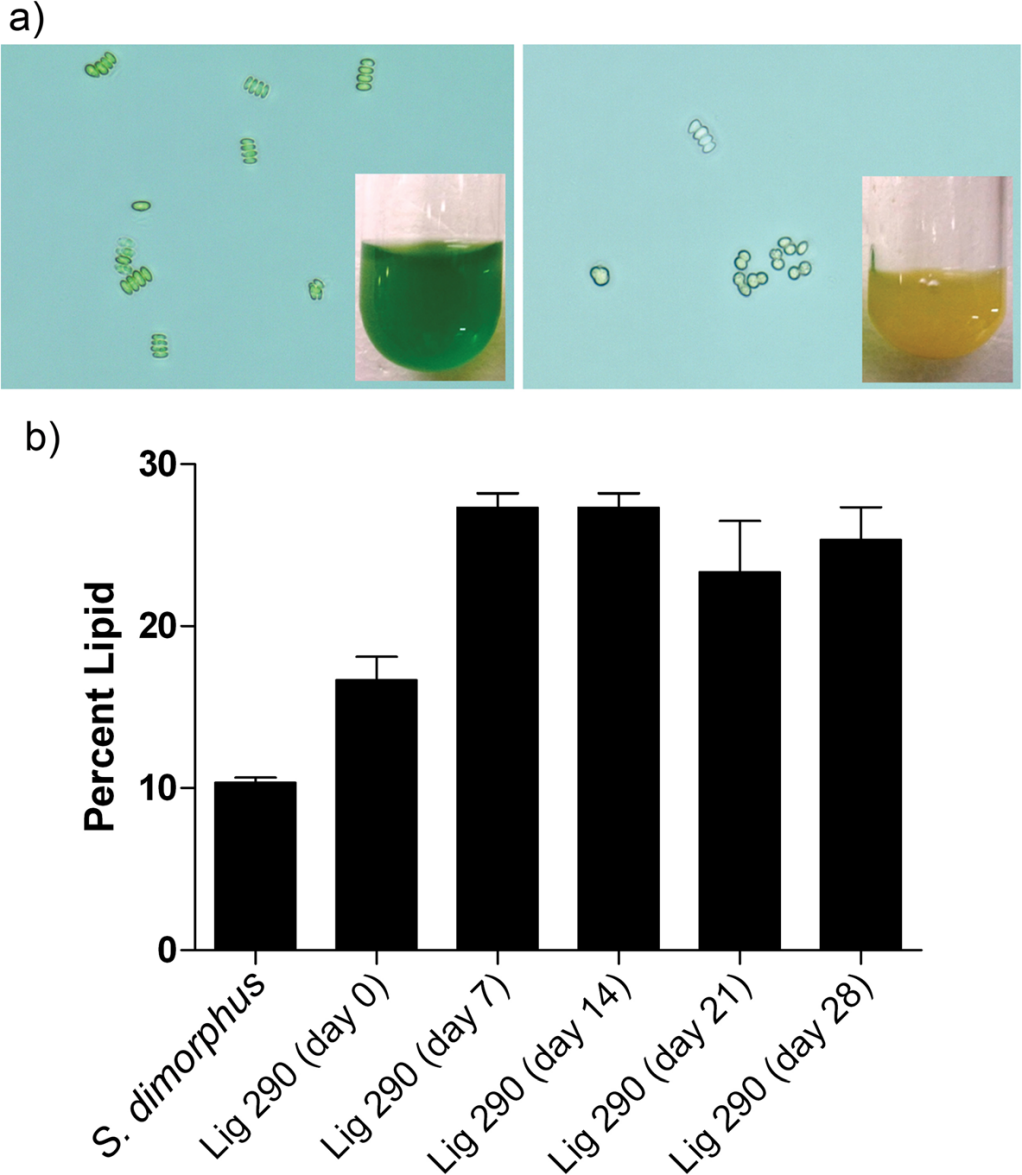
**Lig 290 is identified as a robust high-lipid- producing *Scenedesmus spp. *a)** When cultured at neutral pH the cells proliferate and maintain a green pigmentation. When cultured under low pH conditions (pH = 3), the microalgae take on a yellow/gold pigmentation. **b)** Gravimetric lipid analysis demonstrates increased in lipid content when cultured at pH = 3 over the course of 7–28 days.

When cultured in low pH conditions (pH = 3) for 14 days, Lig 290 underwent a physiological shift changing in color from green (chlorophyll rich) to gold (lipid rich) color as demonstrated in the panel insert of Figure 4a. We also observed a change in morphology where *Scenedesmus spp.* quartet coenobia disassociated into a unicellular form that we ascribe to a stressed state*.*

To assess if Lig 290 is indeed a high-lipid producing species of algae, triplicate total lipid content was analyzed as described in by Folch et al. ( 1957). Lig 290 grown in log phase indicated a relative lipid content of approximately 16% when cultured at pH 7 as compared to 27% when cultured at pH 3. Comparatively, the laboratory strain of *Scenedesmus dimorphus* demonstrated a lipid content of approximately 10%, in laboratory conditions (pH 7) over the same period (Figure 4b).

**Figure 5:**
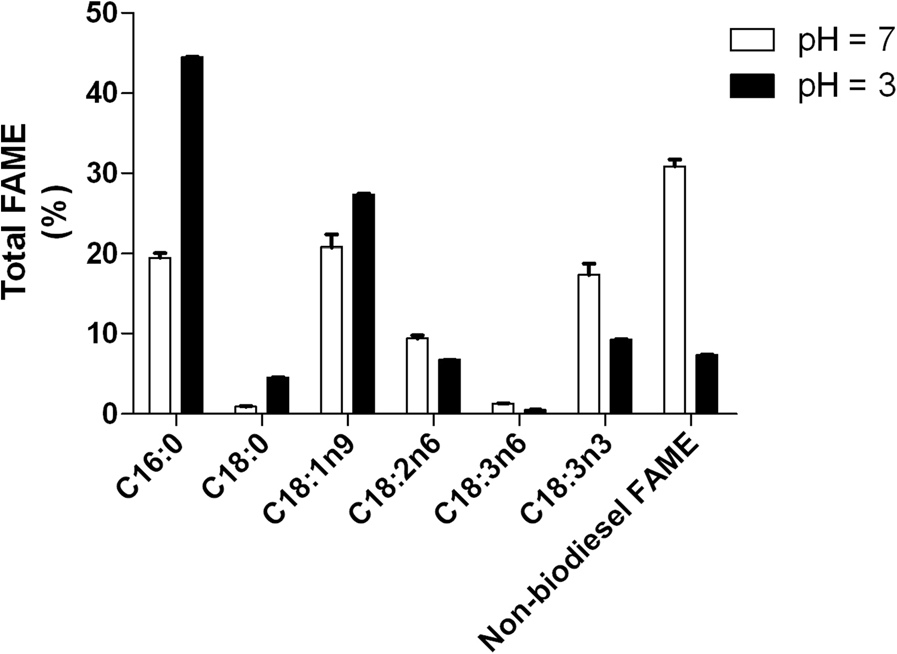
**Lipid characterization of Lig 290.** Lipid were extracted and derivatized to fatty acid methyl esters (FAME) and characterized via gas chromatography. The FAME speciesare consistent with a desirable biodiesel profile, categorized as C16 (C16:0–2), C18(C18:0–2), and C20 (which were not present). Omega-3′s contain C16 or C18 species. All other FAMEs were classified as non-biodiesel. Results presented are mean values (n = 3) and error bars are the standard error of the mean.

In order to confirm that the lipids produced by Lig 290 are appropriate for use as biodiesel, we performed a direct transesterification reaction. Upon evaporating excess organic solvent, a methanol-based transesterification was performed to convert Lig 290 isolated fatty acids to fatty acid methyl esters (FAME). FAMEs were then analyzed using gas chromatography, equipped with a flame ionization detector. Manual integration of the peak areas indicated that culturing at a pH 3 resulted in over 90% of the FAMEs produced by Lig 290 classified as high quality biodiesel (C16 – C18) with a single species of C16 FAME (C16:0) and multiple species of C18 (Figure 5). Additionally, the low pH condition also resulted in a dramatic increase in the amount of C16:0 FAME content. These results suggest that lower pH induces a shift in the FAME profile of Lig 290 towards biofuel compatible lipids. In addition to FAMEs, Lig 290 also produced a number of polyunsaturated fatty acids.

## Discussion

We propose that a strain isolated from an acidified water body in proximity to an abandoned lignite mine, Lig 290, may have potential advantages to *Scenedesmus* strains which are currently being exploited for biofuel production. Based on our characterization of Lig 290, this organism can yield considerably higher lipid production rates as compared to *Scenedesmus dimorphus*. But equally importantly, as Lig 290 was isolated from a low pH water body, it has a greater probability of maintaining a robust growth profile in conditions that will discourage invasive species in a commercial production setting or for culturing in acidified environments (e.g. mine tailing pond).

In general, the use of microalgae for biofuel production relies upon the exploitation of a collection of well-characterized laboratory strains (Chisti 2007). As a result, the field lacks a diversity of robust strains which can thrive in conditions that would discourage non-lipid productive invasive species. Therefore, the identification of versatile cultures derived from extreme environmental sources, has the potential to substantially increase the efficiency and economic viability of microalgal-based green energy resources.

We chose a lipophilic screening approach using Nile Red staining. While the Nile Red screening strategy has the advantage of being high throughput, it is possible that some interesting strains could be missed due to the fact that cell wall composition, cell size and trophic levels in the sample negatively influence the number of positive hits (Cirulis et al. 2012). It should also be noted that Lig 290 also demonstrated more productive growth at pH 4 as compared to pH 3. At the lower pH condition we observed a physiological shift from a green morphology to a gold morphology within fourteen days. This phenomena was not observed at pH 4 or pH 7. We interpret this to indicate that while Lig 290 is acidophilic, pH 3 may be outside optimal growth conditions and may result in a decrease of the observed growth rate, and/or simultaneous pheophytinization of the chlorophyll. Thus, optimal growth conditions for Lig 290 occurred at a pH 4 and greater.

Numerous studies have examined the implications of microalgae upon the production of value-added bio-products (Carvalho and Malcata 2005; Cohen et al. 1995; Doughman et al. 2007; Zhou et al. 2012). In particular, there has been growing interest in the use of microalgae as an alternative source of omega-3/6 fatty acids due to a growing awareness of the potential health benefits of these polyunsaturated fatty acids (Carvalho and Malcata 2005; Cohen et al. 1995; Doughman et al. 2007; Zhou et al. 2012). The use of versatile and robust microalgae strains can, therefore, present additional benefits beyond the implementation of their use in biodiesel production. As evidenced by our gas chromatography analysis of Lig 290, the lipid profile was altered to include a greater abundance of polyunsaturated fatty acids (PUFA’s) (Figure 5). Therefore, the economic feasibility of large-scale production of microalgae may be enhanced by considering co-production of biodiesel and nutraceuticals.

Potentially, the most valuable application for Lig 290 would be in an industrial process-coupled production setting. Several industrial processes produce excess amount of carbon dioxide. This waste product has the potential to be repurposed as a feedstock for microalgae. In the mining sector, off-gas that is typically exhausted to the environment could be sparged into culture vessels to provide agitation and CO_2_ as a carbon source for photosynthesis and lipid production (Scott et al., 2010). A technical challenge to this potential process is potential drop in culture pH as smelting off-gas is typically rich in sulfur dioxide (Shang and Scott 2011), which in an aqueous environment can result in the production of sulfuric acid. However, due to ability for Lig 290 to survive at a low pH, this acid-tolerant microalgae could conceivably consume CO_2_ in the off-gas and convert it to biomass (Larkum et al. 2012).

A considerable amount of work has been done to identify marine microalgae for the purpose of biofuel production, but freshwater microalgae can also be of use in future development of bioenergy production (Danielewicz et al. 2011; Do Nascimento et al. 2012; Lim et al. 2012; Takagi et al. 2006). For example, mining activities tend to render large tracts of land and fresh water ponds in a brown water state. Exploiting these ponds for the growth of microalgae could conceivably produce large yields of carbon-neutral biofuel as a byproduct of mining activities. Thus, the continued identification and characterization of novel strains of microalgae, such as the *Scendesmus spp.* Lig 290, may have future application for several green energy applications.

Future efforts to improve selective culturing strategies may benefit from the identification of novel microalgae strains. To date, efforts have focused on well characterized laboratory strains, but several groups have begun to explore bioengineered strains which have many desirable characteristics (Rosenberg et al. 2008). While recombinant technologies offer interesting opportunities, this study demonstrates that indentifying new algal strains from stressed environments may be a worthwhile and complementary strategy to further the ability to produce third generation biofuels and value-added products more effectively.

As carbon sequestration and biofuel technologies mature, microalgae-based feedstock will continue to be a critical aspect of the process productivity. Most algal strains currently used in open raceway biofuel production are derived from laboratory settings. A major challenge for this culturing strategy is competition from regional invasive species. By using strategies similar to those presented in this study, the identification of novel strains which, not only demonstrate robust growth and high lipid production, but are also capable of growing in selective culturing conditions may decrease the frequency of culture contamination and batch loss. Lig 290 may serve as a model strain for evaluating acidophilic culturing strategies.

## Competing interests

The authors declare that they have no competing interests.

## Authors’ contributions

JKE contributed to sampling, experimentation, experimental design and manuscript preparation. JDC performed growth and lipid screening experiments. GNS performed environmental characterization and strain purification/identification. KZ performed gas chromatography and gravimetric lipid analysis. NSH, JM and CL performed growth profiling experimentation. CL and JAS contributed to experimental design and manuscript writing. GMR contributed to experimental design, manuscript writing and environmental sampling. All authors read and approved the final manuscript.
